# The heart’s exposure to radiation increases the risk of cardiac toxicity after chemoradiotherapy for superficial esophageal cancer: a retrospective cohort study

**DOI:** 10.1186/s12885-019-5421-y

**Published:** 2019-03-04

**Authors:** Yoshito Hayashi, Hideki Iijima, Fumiaki Isohashi, Yoshiki Tsujii, Tetsuji Fujinaga, Kengo Nagai, Shunsuke Yoshii, Akihiko Sakatani, Satoshi Hiyama, Shinichiro Shinzaki, Tomoki Makino, Makoto Yamasaki, Kazuhiko Ogawa, Yuichiro Doki, Tetsuo Takehara

**Affiliations:** 1 Department of Gastroenterology and Hepatology, Osaka University Graduate School of Medicine, 2-2, Yamadaoka, Suita, Osaka, 565-0871 Japan; 2 Department of Radiation Oncology, Osaka University Graduate School of Medicine, 2-2, Yamadaoka, Suita, Osaka, 565-0871 Japan; 3 Department of Gastroenterological Surgery, Osaka University Graduate School of Medicine, 2-2, Yamadaoka, Suita, Osaka, 565-0871 Japan

**Keywords:** Chemoradiotherapy, Superficial esophageal cancer, Organ preservation, Late toxicity, Cardiac disease

## Abstract

**Background:**

Chemoradiotherapy effectively treats superficial esophageal cancer and is optimal to preserve organs. However, late toxicity, particularly in cardiac diseases, obstructs clinical outcomes. We revealed the risk factors for cardiac event development post-chemoradiotherapy.

**Methods:**

Data from 80 patients who were diagnosed with submucosal invasive esophageal cancer without metastasis (confirmed using multiple modalities) and who underwent chemoradiotherapy between 2006 and 2014 were analyzed. Patients were 11% (9/80) female, and the median age and follow-up were 66.5 y and 73 mo, respectively. We calculated the individual radiation dose to the heart and analyzed relationships between the cardiac event occurrence rate and each clinical factor.

**Results:**

The 5-y overall and recurrence-free survival rates were 74.6 and 62.4%, respectively. Among the total number of deaths, 34.6% was caused by esophageal cancer. During the follow-up, 13 patients developed severe cardiac events (ischemic heart diseases, *n* = 7; pericardial effusion, *n* = 3, atrial fibrillation, *n* = 1; and sudden death, *n* = 2). The significant risk factor for cardiac events post-chemoradiotherapy was the level of the heart’s exposure to radiation, with higher exposure associated with greater occurrence. History of smoking, obesity, comorbidity, and history of cardiac disease were unrelated to cardiac event occurrence post-chemoradiotherapy.

**Conclusions:**

Chemoradiotherapy is a favorable intervention for superficial esophageal cancer. Reducing the radiation dose to the heart likely contributes to preventing cardiac toxicity post-chemoradiotherapy.

**Electronic supplementary material:**

The online version of this article (10.1186/s12885-019-5421-y) contains supplementary material, which is available to authorized users.

## Background

Today, malignant disease remains one of the largest threats to mankind. Malignant diseases are treated with both enhanced efficacy and reduced invasiveness. Esophageal cancer (EC) is one of the malignancies for which the efficacy and invasiveness of treatment need to be balanced. Esophagectomy remains the standard treatment for invasive EC; however, esophagectomy has a high burden on patients. In addition, the number of elderly patients and patients at high risk of developing comorbidities is increasing. Recently, in an effort to reduce invasiveness without abrogating efficacy, combined modality therapy, such as endoscopic treatment, radiotherapy, and chemotherapy, has been developed. Less invasive interventions have improved, and multiple strategies can be applied to patients with EC according to their individual conditions.

Regarding submucosal invasive EC without lymph node metastasis, surgical resection alone is the standard treatment. Many researchers have evaluated the efficacy of chemotherapy, radiotherapy, and chemoradiotherapy (CRT) to avoid reducing the patient’s quality of life, as well as complications after esophagectomy. Various studies have investigated the efficacy of CRT in the treatment of patients with submucosal invasive EC [1–3]. Based on these results, the number of patients treated with CRT for EC will increase in the future. In particular, CRT will be an alternative choice for elderly patients or those at high risk for developing comorbidities because of its minimal invasiveness.

Consistent with other studies [1, 5], we previously reported that the short-term efficacy of CRT to treat submucosal invasive EC without lymph node metastasis was not inferior to that of esophagectomy [4]; thus, definitive CRT is an option to treat cT1N0M0 stage IA EC. On the contrary, the risk of developing late toxicity, such as pneumonitis, pleural effusion, and pericardial effusion, is an issue that needs to be resolved. We previously investigated the risk factors of pericardial effusion that were greater than Grade 1 (according to the Common Terminology Criteria for Adverse Events (CTCAE), version 4.0) in patients with intramucosal and submucosal EC who underwent definitive CRT [6]. However, the risk factors for developing severe cardiac toxicity remain unknown, although clarifying these risk factors may improve the outcomes from definitive CRT for submucosal EC. Therefore, the present study aimed to elucidate the risk factors that led to cardiac events (CEs) after CRT for EC with submucosal invasion.

## Methods

### Patients

We retrospectively analyzed data from 80 patients with EC who were treated with CRT at Osaka University Hospital between 2006 and 2014, and were subsequently followed for more than 2 y. All patients were pathologically diagnosed with squamous cell carcinoma via endoscopic biopsy. The clinical stage was evaluated using a magnified endoscopy with narrow band imaging, iodine staining, endoscopic ultrasonography, chest and abdominal computed tomography (CT), and positron emission tomography-CT (PET-CT). All patients were diagnosed with cT1 (submucosal invasion), N0, M0, stage IA, according to the Eighth Edition of the Union for International Cancer Control (UICC) TNM Classification of Malignant Tumors; patients with intramucosal EC were not included. Clinical factors were collected to analyze their relationship with CE. Data on age, sex, body mass index, tumor location, history of heart disease, smoking habit, and medication for abnormal lipidemia, diabetes mellitus, and hypertension were collected. We compared these factors between patients who did or did not experience CEs after CRT.

### Definitive, concurrent chemoradiotherapy

All patients satisfied the following criteria: 1) age over 20 y, 2) Eastern Cooperative Oncology Group performance status of 0 or 1, 3) patients whose principal organs functioned normally, 4) those with no history of radiation to the chest, 5) and those who provided written informed consent. Chemotherapy consisted of 5-fluorouracil (5-FU) and cisplatin (CDDP), according to the protocol of a previous study [1]. The 5-FU was administered continuously at a dose of 700 mg/m^2^ on days 1–5 and 29–33 (at 24 h and 4 d, respectively). CDDP was administered at a dose of 70 mg/m^2^ on days 1 and 29. In cases with renal dysfunction, we used nedaplatin instead of CDDP. Radiotherapy was performed at 60 Gy with 30 fractions on weekdays that were concurrent with chemotherapy. The dose distribution was calculated using a commercial treatment planning system (XiO; Elekta, Stockholm, Sweden) with superposition algorithm. Radiotherapy was delivered by 10 MV photons from a linear accelerator. The clinical target volume (CTV) was determined according to the esophageal tumor location plus 1–2 cm, which was marked using endoscopic clipping, before simulation CT. The planning target volume was defined as CTV plus the 1–2 cm margin. Irradiation was performed until 40 Gy was reached using anterior-to-posterior opposing fields. Then, irradiation was performed using obliquely opposing fields to avoid the spinal cord until 60 Gy was reached.

### Surveillance

Chest and abdominal CT scans and endoscopy were performed every 3 mo for the first 12 mo after initiation of the treatment, and then every 6 mo thereafter. PET-CT was performed, as needed. Metachronous EC that was detected outside of the radiation field was not defined as recurrence; however, metachronous mucosal cancer within the radiation field that was removed via endoscopic treatment was considered as recurrence in this study. Salvage treatment for recurrence was not prescribed.

### Cardiac events and analysis of radiation dose to the heart

CE were defined as ischemic heart diseases, arrhythmia, pericardial effusion, and sudden death that were categorized as Grade 3 or higher based on the CTCAE, version 4.0. Cardiologists intervened as soon as cardiac diseases occurred. Sudden death was defined as a death with an unknown cause. The radiation dose to the heart was retrospectively evaluated by two radiologists using multi-detector CT scans in order to plan the treatment. The surface of the heart, which was defined as the inferior border of the right pulmonary artery to the apex of the heart, was manually delineated on each axial CT slice. We calculated the dose-volume parameters of the heart, including the whole heart volume, maximum dose (Dmax), minimum dose (Dmin), mean dose, and the percentage or the absolute volume of the heart’s exposure to irradiation more than 1500, 3000, 4000, and 5000 cGy (V1500–5000 cGy [%] or V1500–5000 cGy [mL], respectively).

### Statistical analysis

Continuous variables are expressed as median and interquartile range, and were analyzed using a *t*-test or Wilcoxon test. Categorical variables were analyzed using a chi-squared test or Fisher’s exact test. For the univariate test, we used a logistic regression model and Cox proportional hazards model. The survival rate and the CE occurrence rate were calculated using the Kaplan-Meier method. *P*-values less than 0.05 were considered statistically significant. All statistical analyses were performed using JMP software (ver. 13.1; SAS Institute Inc., Cary, NC, USA).

## Results

### Patients

The characteristics of the patients are shown in Table 1. All but two patients completed the treatments successfully. One patient received only 52 Gy irradiation because he wished to discontinue the treatment, and one patient received an 80% dose of chemotherapy in the later part of the treatment course because of a digestive adverse event. The median follow-up duration for censored cases was 73 mo.

**Table 1:** Characteristics of patients and tumors

Factors	*n* = 80
Age (y)	66.5 (62–73)
Sex (male/female)	71/9
Main tumor location (Ce-Ut/Mt./Lt-Ae)	16/46/18
Diameter (mm)	40 (20–50)
Macroscopic findings (elevated/flat-depressed)	18/62
History of cardiac diseases (yes/no)	5/75

Data are presented in median (interquartile range) or number. Ce, cervical esophagus; Ut, upper thoracic esophagus; Mt., middle thoracic esophagus; Lt, lower thoracic esophagus; Ae, abdominal esophagus

**Fig. 1:**
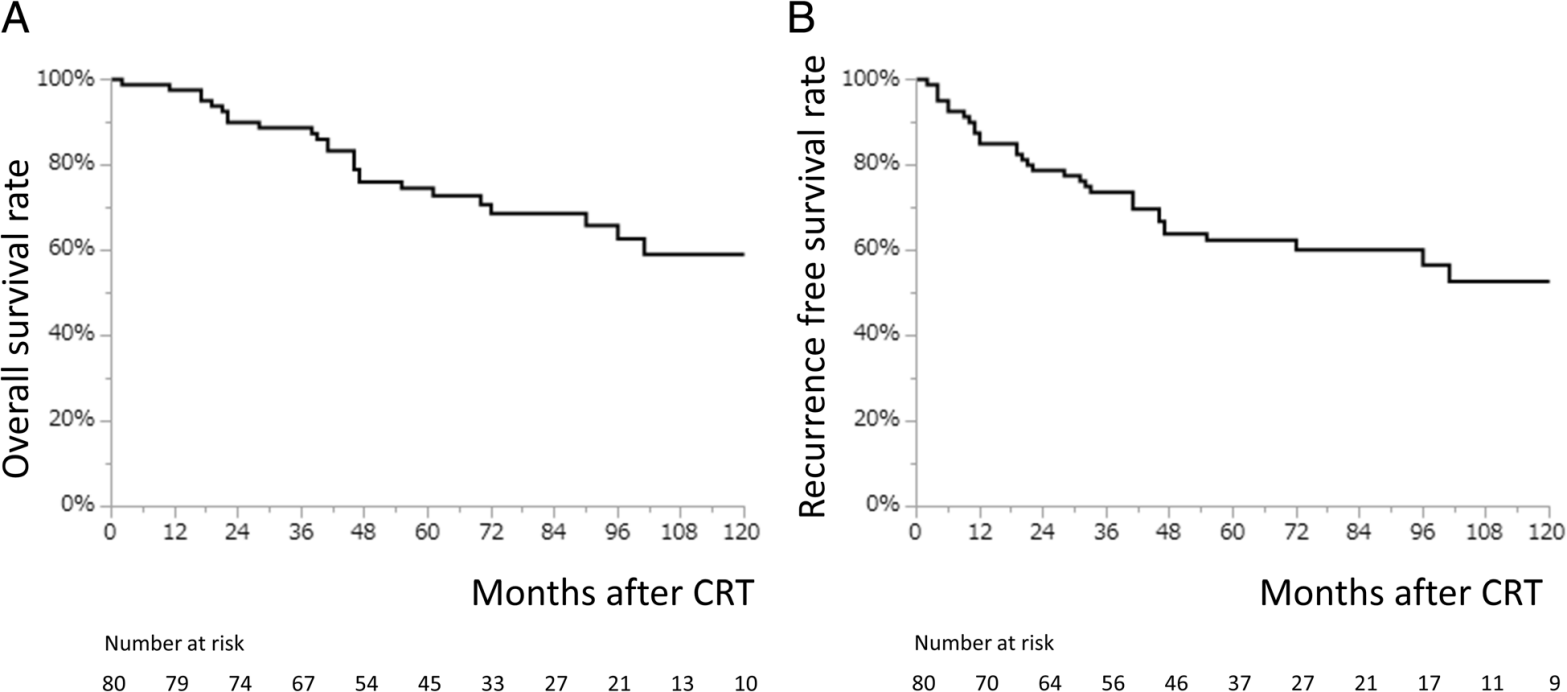
Patient survival rates The overall survival rate (**a**) and the recurrence-free survival rate (**b**). CRT, chemoradiotherapy

The long-term outcomes from CRT are presented in Fig. 1. Of note, the 5-y overall and recurrence-free survival rates in our cohort were 74.6% (95% confidence interval [CI], 63.5–83.2) and 62.4% (95% CI, 51.1–72.6), respectively. Overall, 26 patients died, 9 (34.6%) of which were EC-related deaths. The other causes of death were other malignancies (*n* = 10), liver cirrhosis (n = 1), pneumonia (n = 1), cardiac diseases (*n* = 3), and sudden death (*n* = 2). It is widely known that synchronous or metachronous malignancies in other organs frequently occur in patients with EC. Regular systemic surveillance is important to prevent deaths caused by other malignancies. However, the significance of CE in patients with EC after CRT remains unknown.

**Fig. 2:**
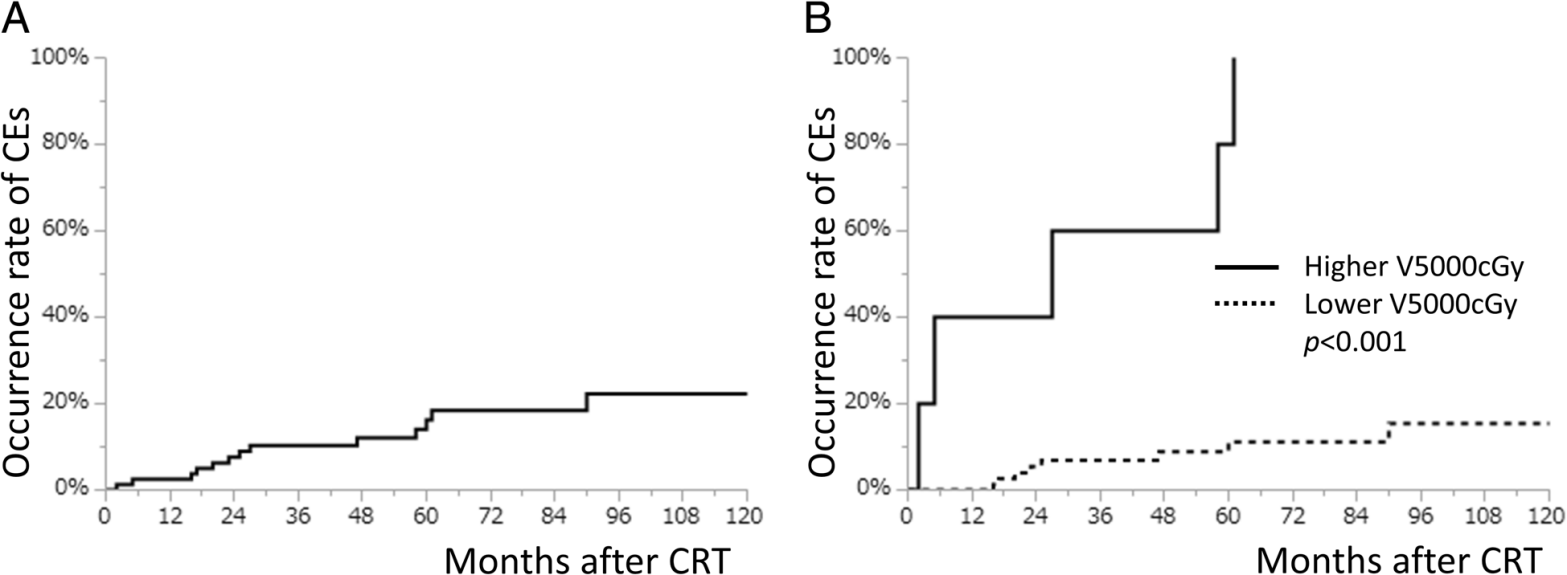
Occurrence rate of cardiac events after chemoradiotherapy Occurrence rates are presented for esophageal cancer in all patients (**a**) and separated by patients with a volume in the heart of higher or less than 280 mL of V5000 cGy (**b**). CE, cardiac events; CRT, chemoradiotherapy

During the follow-up period, 13 patients developed severe CEs (seven ischemic heart diseases, three pericardial effusions, one atrial fibrillation, and two sudden deaths). The occurrence rate of CEs is presented in Fig. 2. Of note, the 5-y cumulative CE occurrence rate was 16.3% (95% CI, 9.1–27.5) (Fig. 2a). These results suggest that preventing CEs would likely improve the clinical outcomes of CRT for EC.

### Classical factors related to ischemic coronary disease were not prognostic for the occurrence of cardiac diseases after chemoradiotherapy

Next, we analyzed the risk factors for developing CEs after CRT. Detailed information on CEs, tumor characteristics, and irradiation data are shown in Additional file 1: Table S1. To reveal the risk factors that led to the development of CEs, we compared the clinical characteristics of patients who experienced CEs during the follow-up period after CRT (*n* = 13) and those without CEs (non-CE, *n* = 67) (Table 2). A smoking habit was one of the most important risk factors for developing a cardiac disease; however, neither the number of cigarettes nor the years of smoking were related to the occurrence of CEs. Comorbidities, such as diabetes mellitus, hyperlipidemia, and hypertension, are well-known risk factors of CEs. The presence of these diseases did not predict the development of CEs after CRT. A history of cardiac diseases also was not associated with the occurrence of CEs after CRT. Additionally, tumor factors, such as macroscopic morphology, diameter, and location, or patient factors, such as age, sex, and body mass index, were similar between CE and non-CE patients.

**Table 2:** Comparison of risk factors for developing cardiac events

	CE (*n* = 13)	Non-CE (*n* = 67)	*P* value
Patients			
Age (y)	66 (60–74)	67 (62–73)	0.5308
Sex (male/female)	13/0	58/9	0.3419
BMI (kg/m^2^)	19.6 (17.8–22.6)	20.9 (18.7–23.1)	0.3894
Tumors			
Tumor location (Ce-Ut/Mt./Lt-Ae)	1/9/3	15/37/15	0.4009
Tumor size (mm)	50 (30–75)	40 (20–50)	0.2255
Macro findings (elevated/flat-depressed)	3/10	15/52	1.0000
Habits			
Smoking frequency (number/d)	20 (20–40)	20 (10–40)	0.1049
Smoking history (y)	33 (25–46)	35 (20–45)	0.5633
Brinkman index	1000 (600–1215)	750 (380–1170)	0.2117
Comorbidity			
Hypertension	6 (46.2%)	30 (52.6%)	1.0000
Diabetes mellitus	2 (15.4%)	8 (14.0%)	0.6624
Hyperlipidemia	1 (7.7%)	10 (17.5%)	0.6817
History of cardiac disease	1 (7.7%)	4 (7.0%)	1.0000

Data are presented as median (interquartile range), number, or number (%). CE, cardiac event; BMI, body mass index; Ce, cervical esophagus; Ut, upper thoracic esophagus; Mt., middle thoracic esophagus; Lt, lower thoracic esophagus; Ae, abdominal esophagus

### The level of the heart’s exposure to radiation was the major prognostic factor for the occurrence of cardiac diseases

We also analyzed the ratio of irradiated volume to the total heart volume and absolute irradiated volume in the heart (Table 3). Maximum and mean irradiated volumes to the heart were significantly larger in CE patients. Notably, the ratio of irradiated to total heart volume and the absolute heart’s exposure to radiation and was significantly higher in CE patients than in non-CE patients.

**Table 3:** Comparison of the heart’s exposure to radiation between patients with and without cardiac events

	CE (n = 13)	Non-CE (n = 67)	*P* value
Heart volume (mL)	679 (583–770)	660 (566–726)	0.5356
Heart Dmax (cGy)	6164 (6124–6261)	6092 (6030–6200)	0.0363
Heart Dmin (cGy)	46 (15–132)	35 (10–80)	0.5393
Heart Dmean (cGy)	3364 (2673–3882)	2920 (1763–3398)	0.0446
Percentage of the heart volume receiving more than indicated irradiation dose			
V1500 cGy (%)	71.9 (55.8–82.6)	65.7 (40.6–75.0)	0.0976
V3000 cGy (%)	61.1 (47.0–70.0)	52.9 (28.9–64.1)	0.0669
V4000 cGy (%)	53.1 (41.3–62.3)	41.9 (20.9–53.4)	0.0266
V5000 cGy (%)	30.1 (20.8–37.2)	19.4 (10.3–28.2)	0.0167
Absolute heart volume receiving more than indicated irradiation dose			
V1500 cGy (mL)	556 (327–627)	411 (272–498)	0.0699
V3000 cGy (mL)	432 (269–536)	341 (203–428)	0.0357
V4000 cGy (mL)	360 (222–468)	255 (142–364)	0.0202
V5000 cGy (mL)	193 (115–294)	134 (66–187)	0.0084

Data are expressed as median (interquartile range). CE, cardiac event; Dmax, maximum dose; Dmin, minimum dose; Dmean, mean dose

To investigate the impact of radiation exposure to the heart, we calculated the area under the receiver operating characteristics curve. Of multiple parameters, such as the percentage of the heart volume or the absolute volume of the heart receiving irradiation, V5000 cGy (mL) was the largest (area, 0.7325). As a result, we evaluated the cut-off value of V5000 cGy (mL) and identified 281.9 mL as its cut-off value. This value had high specificity (100%), positive predictive value (100%), and negative predictive value (89%), but poor sensitivity (38%). We further categorized the patients into two groups based on whether their volume in the heart was higher (*n* = 5) or lower (*n* = 75) than 280 mL of V5000 cGy (mL). Cox proportional hazards model revealed that the risk ratio was 16.80 (95% CI, 4.94–53.07). The occurrence rate of CEs after CRT was significantly higher in patients exposed to radiation with more than 280 mL of V5000 cGy (mL) than in those exposed to less than 280 mL of V5000 cGy (mL) (Fig. 2b).

## Discussion

The present study demonstrated that the 5-y survival rate of CRT used to treat submucosal EC was 74.6%. The 5-y survival rate of surgical esophagectomy for patients with stage I EC (identified using the sixth edition of the UICC TNM classification), which included both intramucosal and submucosal cancers, was 77.5% in Japan [7]. Therefore, the efficacy of CRT was considered equivalent to that of surgery. Our results also suggest that the amount of the heart’s exposure to radiation is a critically important prognostic factor of CE after CRT. Preventing not only the recurrence of primary disease, but also the occurrence of cardiac diseases or sudden death is important to improve the outcome of CRT. A previous Japanese survey reported that the prevalence rate of myocardial infarction was 0.8% for men and 0.2% for women aged 60 years [8]. Regarding atrial fibrillation, the prevalence rates were 1.3–3.2% for men and 0.5–0.9% for women aged 60 years [8, 9]. The present study revealed that the radiation dose to the heart was involved in cardiac toxicity after CRT for patients with EC.

In recent years, the significance of cardiac toxicity in patients with certain types of malignancies who received anti-cancer therapy has remarkably increased [10]. Accordingly, preventing cardiovascular events is important for cancer survivors [11]. Particularly, cardiac toxicity is more evident in patients with breast cancer compared to patients with EC. Many therapeutic regimens, such as radiation, chemotherapy, molecule-targeted agents, endocrine agents, and anesthetic procedures, are involved in the development of cardiac toxicity during treatment for breast cancer [12]. Concurrent or sequential treatment with radiation and chemotherapy might have a synergistic effect on cardiac toxicity. A meaningful increase in the risk of cardiovascular death was observed in patients who received irradiation for left breast cancer with long follow-up [13]. Radiation-induced CEs are caused by micro- and macro-vascular damages [14]. Vascular injuries cause myocardial damage, arrhythmias, and pericardial effusion. The pathology and mechanism of coronary artery damage due to radiation seem to be similar to those of classical coronary diseases.

In past decades, the role of radiotherapy for EC has increased in importance [15]. As the treatment efficacy improves, the incidence of late toxicity after CRT for EC has increased. The risk factor of cardiopulmonary toxicity of Grade 3 or higher after CRT for advanced EC was reported in patients older than 75 y, who had no concurrent history of smoking or cardiopulmonary disease [16]. However, cardiac toxicity is an issue that needs to be resolved to treat superficial EC. The risk factors of cardiac toxicity after CRT for EC remain unknown. Typically, smoking and alcohol are well-known risk factors for EC. On the contrary, smoking, diabetes mellitus, and hyperlipidemia are risk factors for cardiac coronary diseases. A potentially high proportion of patients with EC might suffer from cardiac diseases; however, the present study did not reveal a relationship between CEs and smoking or comorbidities, such as diabetes mellitus, hyperlipidemia, and hypertension. Additionally, a history of cardiac diseases was not associated with the occurrence of CEs after CRT.

Combined 5-FU and CDDP remains the standard chemotherapy regimen for treating EC [17]; however, each of these may cause CEs [18]. Furthermore, the risk of complications increases when 5-FU and CDDP are combined [19]. Our study could not elucidate the risk of combined chemotherapy or the synergistic effect of radiotherapy and chemotherapy, because chemotherapy or radiotherapy alone is not performed in practice. By optimizing radiotherapy to diminish cardiac toxicity, two factors are considered: 1) the extent of radiation fields and 2) the amount of radiation dose. There is no international consensus for the definition of radiation field and dose [20]. Prior to CRT, the radiation dose was calculated via radiation planning. The present study suggested that considering the absolute volume of irradiation in the heart or the percentage of irradiated volume to the heart was important to suppress CE after CRT. V5000 cGy (mL) may be a useful predictor for CE. We also evaluated the cut-off value for V5000 cGy (%), and identified 30.09% as the cut-off value. Sensitivity, specificity, positive predictive value, and negative predictive value were 53.9, 82.1, 36.8, and 90.2%, respectively. The significance was less compared to V5000 cGy (mL). It remains unexplained that the absolute irradiated volume is superior to predict CEs compared to the relative irradiated volume against the whole heart volume. Today, three-dimensional conformal radiation therapy is standardly used, and intensity modulated radiation therapy is used for complicated cases in our hospital. The improvements of these modalities would be useful for CE inhibition by avoiding the irradiation against the surrounding organs.

This study had a couple limitations that may have impacted our conclusions. First, this was a retrospective, single-center study and low sample size. Second, cardiac functions prior to CRT, including cardiac ultrasonography and brain natriuretic peptide examination, were not evaluated. Risk assessment for CE occurrence prior to CRT might be important. However, surveillance for CE post CRT might be useful for clinical outcome of patients. Third, there is the gap in the administration during past 10 years regarding with the innovation of tomography.

## Conclusions

Considering the efficacy of CRT and the physical burden of surgery, CRT should be considered as a treatment strategy for patients with invasive submucosal EC. Additionally, we suggest that surveillance for cardiac diseases after CRT is important. Further investigations including cardiac functional conditions prior to CRT are needed. A treatment and surveillance strategy that considers the radiation dose to the heart will improve the survival of patients with invasive submucosal EC.

## Additional file

Additional file 1: **Table S1.** Characteristics of patients with Grade 3–5 cardiac events (DOCX 18 kb)

## Abbreviations

5-FU: 5-fluorouracil
CDDP: Cisplatin
CE: Cardiac event
CRT: Chemoradiotherapy
CT: Computed tomography
CTCAE: Common Terminology Criteria for Adverse Events
CTV: Clinical target volume
Dmax: Maximum dose
Dmin: Minimum dose
EC: Esophageal cancer
JCOG: Japanese Clinical Oncology Group
PET-CT: Positron emission tomography-computed tomography
UICC: Union for International Cancer Control
