# Supplementary material for: The heart’s exposure to radiation increases the risk of cardiac toxicity after chemoradiotherapy for superficial esophageal cancer: a retrospective cohort study
Source: BMC Cancer. 2019 Mar 4;19:195. doi: 10.1186/s12885-019-5421-y (PMC6399839; doi:10.1186/s12885-019-5421-y)
Supplement: Supplementary file 1 — Table S1. Characteristics of patients with Grade 3–5 cardiac events (DOCX 18 kb) [file 12885_2019_5421_MOESM1_ESM.docx]

Supplementary Table 1. Characteristics of patients with grades 3-5 cardiac events

|  | Cardiac events | Onset  (mo) | Range of Age  (yrs) | Loc | Size (mm) | Heart  volume  (mL) | Value of irradiation to heart | | | Ratio of heart’s exposure to radiation | | | | Volume of heart’s exposure to radiation | | | |
| --- | --- | --- | --- | --- | --- | --- | --- | --- | --- | --- | --- | --- | --- | --- | --- | --- | --- |
|  |  |  |  |  |  |  | Mean  (Gy) | Max  (Gy) | Min  (Gy) | V1500  cGy (%) | V3000  cGy (%) | V4000  cGy (%) | V5000  cGy (%) | V1500  cGy (mL) | V3000  cGy (mL) | V4000  cGy (mL) | V5000  cGy (mL) |
| 1 | Sudden death | 47 | 60s | Lt | 20 | 546 | 3363 | 6150 | 30 | 70.96 | 60.88 | 53.13 | 30.24 | 387 | 332 | 290 | 165 |
| 2 | Sudden death | 2 | 70s | Mt | 120 | 1243 | 2833 | 6108 | 32 | 60.31 | 48.95 | 42.61 | 23.51 | 750 | 608 | 530 | 292 |
| 3 | ICD | 61 | 60s | Mt | 50 | 740 | 4313 | 6460 | 230 | 89.26 | 78.21 | 71.62 | 40.12 | 661 | 579 | 530 | 292 |
| 4 | ICD | 60 | 60s | Ut | 50 | 778 | 1507 | 5960 | 0 | 34.18 | 26.33 | 19.78 | 9.35 | 266 | 205 | 154 | 72 |
| 5 | ICD | 27 | 60s | Lt | 30 | 965 | 3329 | 6461 | 46 | 68.2 | 56.88 | 49.88 | 31.81 | 658 | 549 | 481 | 307 |
| 6 | ICD | 25 | 60s | Mt | 40 | 615 | 3243 | 6140 | 40 | 71.92 | 61.06 | 51.04 | 21.78 | 442 | 375 | 313 | 133 |
| 7 | ICD | 23 | 70s | Lt | 40 | 760 | 3643 | 6080 | 60 | 78.24 | 68.64 | 58.57 | 30.09 | 595 | 522 | 445 | 228 |
| 8 | ICD | 17 | 70s | Mt | 50 | 367 | 2513 | 6191 | 0 | 51.38 | 45.03 | 39.45 | 19.89 | 188 | 165 | 144 | 73 |
| 9 | ICD | 16 | 70s | Mt | 15 | 646 | 1643 | 6170 | 0 | 34.95 | 27.19 | 22.77 | 14.71 | 226 | 175 | 147 | 95 |
| 10 | Pericarditis | 91 | 70s | Mt | 110 | 561 | 3717 | 6180 | 50 | 76.26 | 68.79 | 61.11 | 34.36 | 428 | 386 | 343 | 193 |
| 11 | Pericarditis | 58 | 50s | Mt | 80 | 605 | 4520 | 6164 | 143 | 91.76 | 83.2 | 75.17 | 48.82 | 555 | 504 | 455 | 295 |
| 12 | Pericarditis | 20 | 70s | Mt | 30 | 678 | 3588 | 6157 | 146 | 82.48 | 63.69 | 53.09 | 27.04 | 559 | 432 | 360 | 183 |
| 13 | AF | 5 | 50s | Mt | 70 | 696 | 4048 | 6330 | 120 | 82.72 | 71.04 | 63.42 | 40.49 | 575 | 494 | 441 | 281 |

Loc, location; Min, minimum; ICD, ischemic coronary diseases; AF, atrial fibrillation; Lt, lower thoracic esophagus; Mt, middle thoracic esophagus; Ut, upper thoracic esophagus.
